# Metastability indexes global changes in the dynamic working point of the brain following brain stimulation

**DOI:** 10.3389/fnbot.2024.1336438

**Published:** 2024-02-19

**Authors:** Rishabh Bapat, Anagh Pathak, Arpan Banerjee

**Affiliations:** Cognitive Brain Dynamics Lab, National Brain Research Centre, Manesar, Haryana, India

**Keywords:** Transcranial Magnetic Stimulation, metastability, Kuramoto model, microstates, complexity, coordination dynamics, whole brain modeling

## Abstract

Several studies have shown that coordination among neural ensembles is a key to understand human cognition. A well charted path is to identify coordination states associated with cognitive functions from spectral changes in the oscillations of EEG or MEG. A growing number of studies suggest that the tendency to switch between coordination states, sculpts the dynamic repertoire of the brain and can be indexed by a measure known as metastability. In this article, we characterize perturbations in the metastability of global brain network dynamics following Transcranial Magnetic Stimulation that could quantify the duration for which information processing is altered. Thus allowing researchers to understand the network effects of brain stimulation, standardize stimulation protocols and design experimental tasks. We demonstrate the effect empirically using publicly available datasets and use a digital twin (a whole brain connectome model) to understand the dynamic principles that generate such observations. We observed a significant reduction in metastability, concurrent with an increase in coherence following single-pulse TMS reflecting the existence of a window where neural coordination is altered. The reduction in complexity was validated by an additional measure based on the Lempel-Ziv complexity of microstate labeled EEG data. Interestingly, higher frequencies in the EEG signal showed faster recovery in metastability than lower frequencies. The digital twin shed light on how the phase resetting introduced by the single-pulse TMS in local cortical networks can propagate globally, giving rise to changes in metastability and coherence.

## 1 Introduction

Processing the complex dynamic environment around us requires flexible exploration of neural coordination states that helps in brain function (Deco and Kringelbach, 2016). The ability to switch between coordination states is driven by the tendency of the dynamical system to traverse through multiple attractors (Haken, 1983; Bressler, 2002; Kelso and Zanone, 2002; Tognoli and Kelso, 2014), quantitatively captured by a mathematical measure, metastability (Tognoli and Kelso, 2014; Deco and Kringelbach, 2016). Reflecting the fundamental role of metastability, modeling studies have found it to be a signature of brain's dynamic core (Deco et al., 2017) and maximized in the resting state (Hellyer et al., 2014; Deco et al., 2017; Naskar et al., 2021; Saha et al., 2023). A plethora of studies further validate this claim by showing changes in metastability to accompany altered or disordered states of consciousness. Metastability is shown to be reduced during loss of consciousness (Jobst et al., 2017; Cavanna et al., 2018), following traumatic brain injury (Hellyer et al., 2015) and in Alzheimer's disease (Córdova-Palomera et al., 2017). Interestingly, metastability is found to be higher among schizophrenics (Lee et al., 2018) and following the use of psychedelic drugs (Carhart-Harris et al., 2014; Lord et al., 2019). Some of these studies also indicate changes in cognitive flexibility caused by reduction in metastability (Hellyer et al., 2015; Córdova-Palomera et al., 2017). Are these changes in metastability idiosyncratic or do they arise from a principled organization of brain network dynamics? Answering this question requires hypothesis driven empirical observation followed up with theoretical understanding of whole-brain network dynamics.

Transcranial Magnetic Stimulation (TMS) is known to cause a phase-reset within the stimulated region (Kawasaki et al., 2014; Pellicciari et al., 2017). This can effectively force the underlying neural to get into a coordinated state at least transiently. From a dynamical systems standpoint, getting into an attractor state will make a high dimensional system low-dimensional and thus lead to a reduction in metastability (Pillai and Jirsa, 2017). Thus, even a single-pulse TMS can lower the metastability of brain dynamics. Hence, metastability might also be used as an index of neural dynamics to describe the effects of a given TMS protocol. Using metastability to contextualize the effects of such stimulation could help explain the variability pervasive in TMS research and help optimize therapeutic TMS protocols for disorders known to have altered metastability. Once, perfected with TMS, similar effects can be studies for more emerging methods of non-invasive brain stimulation such as transcranial Direct Current Stimulation (tDCS) and transcranial Alternating Current Stimulation (tACS).

In the present article we test the hypothesis that metastability which is typically associated with the resting state is reduced in a time-window that is time-locked to the onset of TMS pulse. The recovery to pre-stimulus levels of metastability will index the temporal window over which the network is perturbed by the stimulation and may vary with the oscillation frequency. Secondly, we illustrate that using a digital twin—a whole-brain network of phase-coupled Kuramoto oscillators connected with a bio-physically realistic connection topology (Cabral et al., 2011; Jirsa et al., 2017)—can shed light on the dynamic principles that are key to such empirical findings. Taken together, the empirical findings and the theoretical approach enhance our understanding of systems level neural mechanisms that unfold following non-invasive brain stimulation.

## 2 Methods

### 2.1 Data collection

Two datasets were obtained from OpenNeuro (https://openneuro.org/) for the purpose of this study. Dataset 1 had 20 healthy participants (six female, 14 male) with a mean age of 30. Data were collected using a 32 channel BrainVision EEG cap with a sampling frequency of 5 kHz. TMS was delivered using a MagStim figure-of-eight coil held at 45 degrees to the mid-saggital line over the scalp hotspot for the left first dorsal interosseous muscle (the right Primary Motor Cortex). Three hundred seconds of resting state, eyes open EEG data were collected from each participant. Six hundred monophasic TMS pulses were then delivered at 120% of the Resting Motor Threshold (RMT) with 5 s between each pulse, EEG recordings were taken simultaneously. Short breaks were taken every 100 pulses or upon subject request (Hussain, 2019).

Dataset 2 had 13 healthy, right handed participants aged between 18 and 85 years. Data were collected using a 64 channel BrainVision EEG cap with a sampling frequency of 20kHz. TMS was delivered using a Nexstim figure-of-eight coil over the right Primary Motor Cortex. Three hundred seconds of resting state data were collected from each participant. Seventy-five monophasic TMS pulses were delivered at 100% of RMT and at 110% of RMT with 5 s between each pulse, EEG recordings were taken simultaneously (Pavon et al., 2022). In both datasets the coil noise was not masked, but participants were provided with earplugs to reduce the disturbance.

### 2.2 Preprocessing

The resting state EEG and TMS-EEG data were pre-processed prior to analysis using custom MATLAB code and the EEGLAB toolbox (Delorme and Makeig, 2004). The remaining analysis was done using custom Python code with the MNE package (Gramfort, 2013) and the SimNIBS toolbox (Saturnino et al., 2019). All code is made available at the associated bitbucket repository (See Data Availability). The resting state EEG data were processed using a combination of Artifact Subspace Reconstruction (Plechawska-Wojcik et al., 2019) and Multiple Artifact Rejection Algorithm (MARA) (Winkler et al., 2011) based independent component rejection. The TMS-EEG data were preprocessed using the Automated Artifact Rejection for Single Pulse TMS Data (ARTIST) pipeline (Wu et al., 2018). All data was downsampled to 1 kHz and bandpass filtered between 1 and 100 Hz prior to analysis.

### 2.3 Measures of metastability

#### 2.3.1 The standard deviation of the Kuramoto Order Parameter

**Figure 1:**
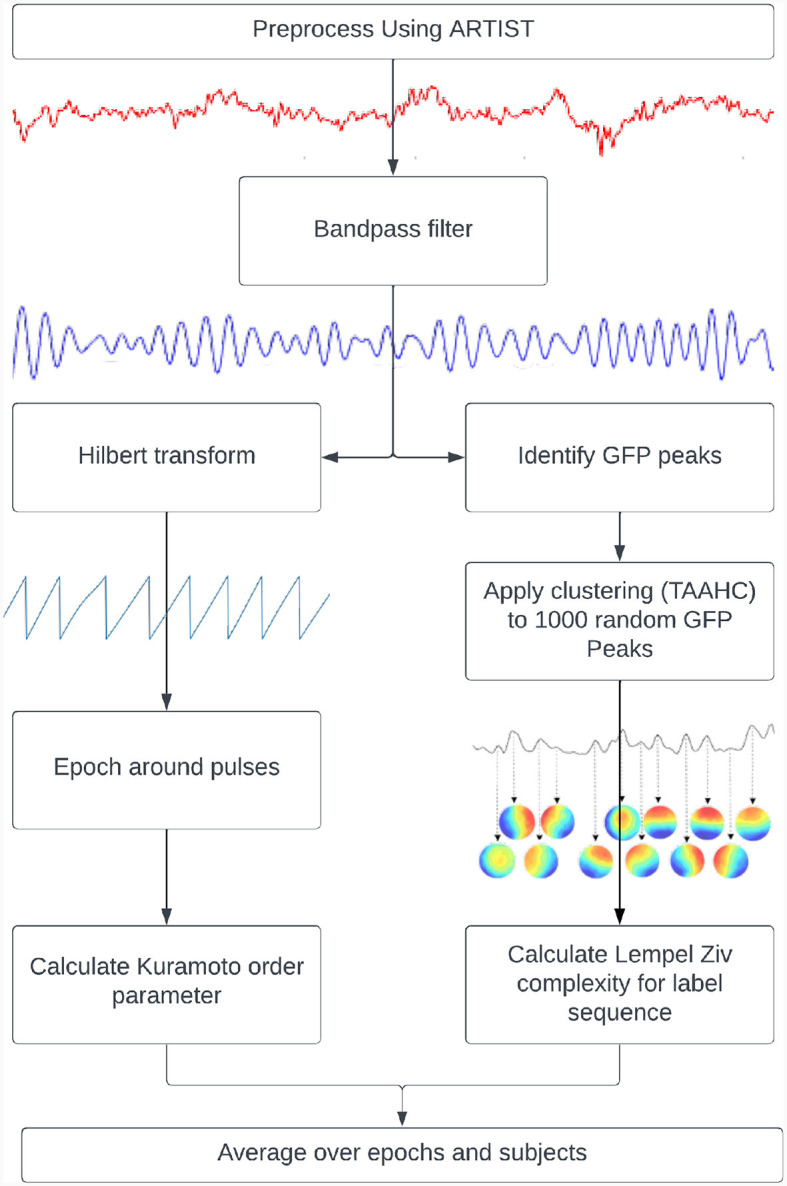
Pipelines used to calculate metastability. Kuramoto Order Parameter on EEG data **(left)** and the Lempel-Ziv Complexity on microstates **(right)**.

Two measures of metastability were applied to the preprocessed EEG time series (Figure 1). The first measure was based on the standard deviation of the Kuramoto Order Parameter (KOP) based on the work of Kuramoto (1984). The KOP is a measure of synchrony in a network of oscillators and is calculated as the real part of the normalized vector sum of individual phases from each node,


(1) $$\begin{array}{l} re^{i\psi }=\frac{1}{N}\overset{N}{\underset{j=1}{\sum }}e^{i\theta_{j}} \end{array}$$

where “*N*” is the number of oscillators, “θ_*j*_” is the phase of the *j*th oscillator, “*r*” is the Kuramoto Order Parameter and ψ is the phase angle following the vector sum of individualized phases. The KOP (Equation 1) can be thought of as plotting the phase of each oscillator as a point on a unit circle, and then taking the magnitude of their resultant vector. Its value is 1 for a completely synchronized system and 0 for a desynchronized system (with infinite nodes). Its standard deviation indexes the ability of the system to deviate from stable states and thus can be used as a proxy measure of metastability. For this measure, the TMS EEG data were bandpass filtered within narrow frequency bands (for example, 8–12 Hz), after which the instantaneous phase was extracted via Hilbert transformation. The KOP was then calculated for each time point. The standard deviation of the KOP was calculated in a sliding window of 50 ms and was used as a dynamic measure of metastability. The metastability time series was then averaged across epochs and participants to yield the final results.

The choice of which group of channels to include for computation of metastability is an important one. Since changes to global coherence across all channels are relatively less intense, and stimulation with TMS has prominent local effects, channel groupings derived from an algorithm were analyzed in addition to global metastability. This algorithm aimed to capture local effects on synchrony and grouped channels together based on the similarity of their TMS evoked potential. First the evoked response to TMS was calculated in each stimulation intensity by averaging across epochs and subjects. Then the time point with the highest Global Field Power (GFP), meaning the highest spatial standard deviation, was selected. At this time point, the activity of each channel was subtracted from the mean of all channels. The channels were then sorted in ascending order of their mean differences, and the top and bottom tertiles were assigned to separate groups. This algorithm is a quantitative analog to visually inspecting the evoked response, finding the point at which the channels activity is the most varied, and sorting the most extreme channels into their own groups.

#### 2.3.2 Microstate sequence complexity

An additional measure of metastability used in this study is based on microstate sequence complexity. Microstates are quasi-stable spatial activity patterns that are derived from EEG data using clustering algorithms. The original recording can then be backfitted to the most similar microstate at each point in time and be analyzed as a sequence of microstates (Lehmann, 1971). The quasi-stable nature of these states is reminiscent of the dwell and escape tendencies seen in metastable dynamics while the microstates themselves are related to the coordination states. The central idea behind this measure is that metastable coordination dynamics will produce a non-repetitive microstate sequence. This can be expressed for any microstate sequence using measures of “complexity.”

“Complexity” refers to the unpredictability of a signal and is quantified here by Lempel-Ziv Complexity (LZC). The LZC of a string is the minimum number of unique sub-strings that can be repeated and combined to reproduce the original. It increases with the unpredictability and length of a string (Lempel and Ziv, 1986). In order to compute this measure, microstates were derived from GFP peaks in resting state data that was bandpassed to alpha (8–12 Hz). The clustering was done using Topographical Atomise and Agglomerate Hierarchical Clustering. In this method, each time point is initially its own microstate. Through iteratively breaking apart (atomising) and redistributing (agglomerating) the worst microstate, based on the sum of correlations between the microstate and its members (Correlation Sum), the number of microstates is reduced to two (a preset minimum) thereafter, an optimal number of microstates can be selected (Khanna et al., 2014; Poulsen et al., 2018).

Microstates were individually created for each participant based on their resting state data. The number of microstates for each participant were selected using the Krzanowski-Lai criterion (Krzanowski and Lai, 1988) applied to the Correlation Sum. This method involves identifying the point past which the Correlation Sum plateaus with respect to the number of microstates. The microstates for each participant were then back fitted to their resting state data, and fitted to their TMS EEG data. After this, the LZC was calculated in a 100 ms sliding window and averaged across epochs and subjects before being compared between resting state and TMS EEG recordings. This short window was considered suitable since good temporal resolution was required for this analysis and previous literature has shown that microstate sequences show scale free dynamics (Van De Ville et al., 2010).

To test the changes in metastability for significance, two 250 ms windows of time were defined, before and after the pulse. Then, the epoch-averaged measures were averaged in the windows of time, yielding a pre and post pulse metastability value for each participant. Given that the sample size was < 30 and the results of the Shapiro–Wilk test of normality were inconsistent, normality could not be safely assumed. Thus the difference between the two lists of metastability measures was tested for significance using Wilcoxon's Signed Rank test. This is a non-parametric test suitable for testing dependent samples. The significance threshold was set to 0.05, and *p*-values below this were used to indicate a significant difference. The test was carried out in a one-tailed manner, with the direction being dependent on the effect in question.

### 2.4 Computational modeling

In order to provide mechanistic insights into metastability modulation post-TMS, a computational model was implemented similar to that used by Pathak et al. (2022). The brain was reduced to a system of 90 coupled oscillators with activity at each oscillator being generated based on the Kuramoto model,


(2) $$\begin{array}{l} \dot{\theta_{i}}=\omega_{i}+\frac{K}{N}*\overset{N}{\underset{j=1}{\sum }}c_{ij}*sin\left(\theta_{j}\left(t-\tau_{ij}\right)-\theta_{i}\right)+d*\zeta \left(t\right) \end{array}$$

Here, the derivative of the phase of each oscillator, “${\dot{\theta }}_{i}$” is calculated using its intrinsic frequency, “ω_*i*_,” a coupling term with a time delay, “τ_*ij*_” and coupling strength, “*c*_*ij*_,” and noise [d*ζ(*t*)]. Euler integration is then used to generate a phase time series for the network. The intrinsic frequencies of the network were assigned based on anatomical node strength, as described in Gollo et al. (2017). The adjacency matrix was derived from probabilistic tractography applied to diffusion MRI data and were obtained from the public repository associated with Cabral et al. (2014). The connectivity was reduced to a 90 by 90 matrix based on the Automated Anatomical Labeling parcellation scheme (Tzourio-Mazoyer et al., 2002) and averaged across subjects as in Cabral et al. (2014). The delays were obtained by scaling cortical distances by conduction velocity. Noise was sampled from a normal distribution with a mean of zero and a standard deviation of 1, it was scaled by a factor of 3. The scaling factors for coupling “*c*_*ij*_” and the conduction velocity that scales “τ_*ij*_” were chosen such that the model showed metastable dynamics, as approximated by the Kuramoto Order Parameter.

Given that TMS is known to cause a phase reset in the affected area, that propagates through the rest of the network (Kawasaki et al., 2014) the TMS pulse was modeled by resetting the phase of affected oscillators to an arbitrary value π/2 (any other value can be chosen for this purpose without loss of generality). During TMS, the stimulated region and nearby areas reset their phase instantly, while other areas would experience the phase reset with a delay as it propagates through the network. To emulate this the region of instant phase resetting was determined by simulating the electric field induced by TMS using SimNIBS (see Supplementary material), while all other regions were reset based on their conduction delay to the site of stimulation. The Kuramoto Order Parameter of the network was found to oscillate reflecting the metastability of the dynamics. To keep the state of the network at the time of stimulation consistent, a peak finding algorithm was used to find the high coherence and low coherence states. The pulse was only delivered at low coherence states. A subgroup of oscillators with similar intrinsic frequencies to the oscillator corresponding to the right primary motor cortex were chosen for further analysis. This subgroup is analogous to the subgroups derived from the empirical data. Metastability was then computed in a 500 ms sliding window based on the KOP. This analysis was repeated for frequency distributions with maximum frequencies of 5, 15, 25, and 35 Hz to observe the effects of intrinsic frequency on the dynamics. The dispersion of the frequency distribution and the other parameters of the model were kept consistent. The metastability and coherence time series were produced for 20 random number generator seeds and averaged.

## 3 Results

### 3.1 Localization of TMS induced neural coordination

**Figure 2:**
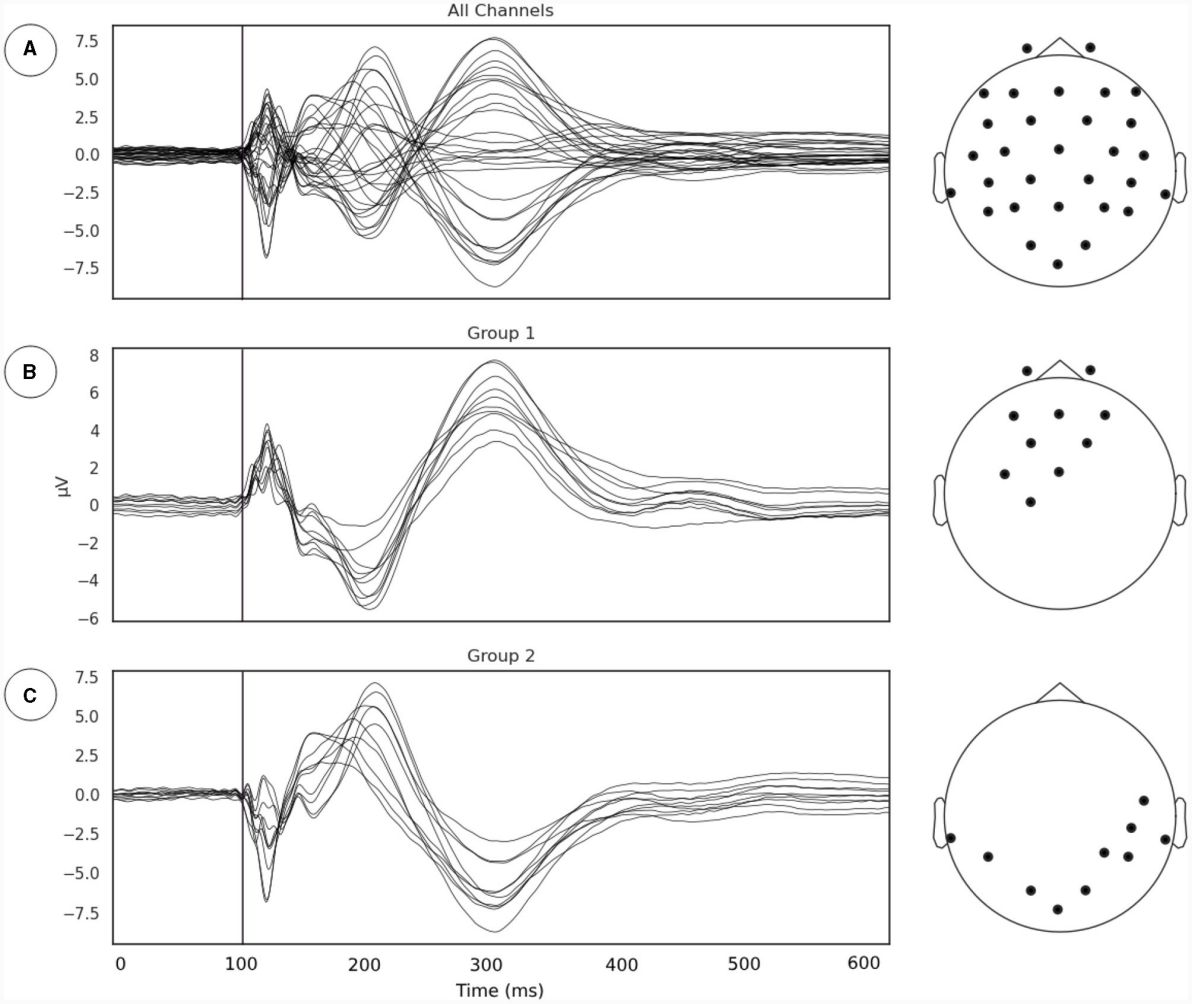
**(A)** Evoked potentials, time locked to onset of TMS pulse. The TMS pulse is indicated by the vertical line. **(B, C)** Channel groupings or the regions of interest for examination derived from measurements of local synchrony.

Our first goal was to identify the subgroup of EEG sensors where the relationship between phase synchrony and metastability is most evident following the line of reasoning outlaid in Section 2.3.1. We extracted the TMS pulse evoked event related potential (ERP) and applied a channel grouping algorithm described in Section 2.3.1 (Figure 2). The channel grouping analysis extracted two distinct groups of sensors, a fronto-central (Figure 2B) and a temporo-occipital (Figure 2C) cluster. Thus, the groups appear to be clustered either around the point of stimulation (right Primary Motor Cortex) and reflected the shape of the induced electrical field.

### 3.2 Metastability in pre and post TMS periods

Metastability was computed using two measures to demonstrate that the phenomena are robust. One measure was based on the Kuramoto Order Parameter (KOP), while the other used Lempel-Ziv complexity based on microstates. While the KOP measures the levels of synchrony in a select group of sensors, the standard deviation of the KOP indexes metastability (Deco et al., 2017; Pathak et al., 2022). The LZC measure is introduced in this study as an alternative measure of metastability (see details in Section 2.3.2).

**Figure 3:**
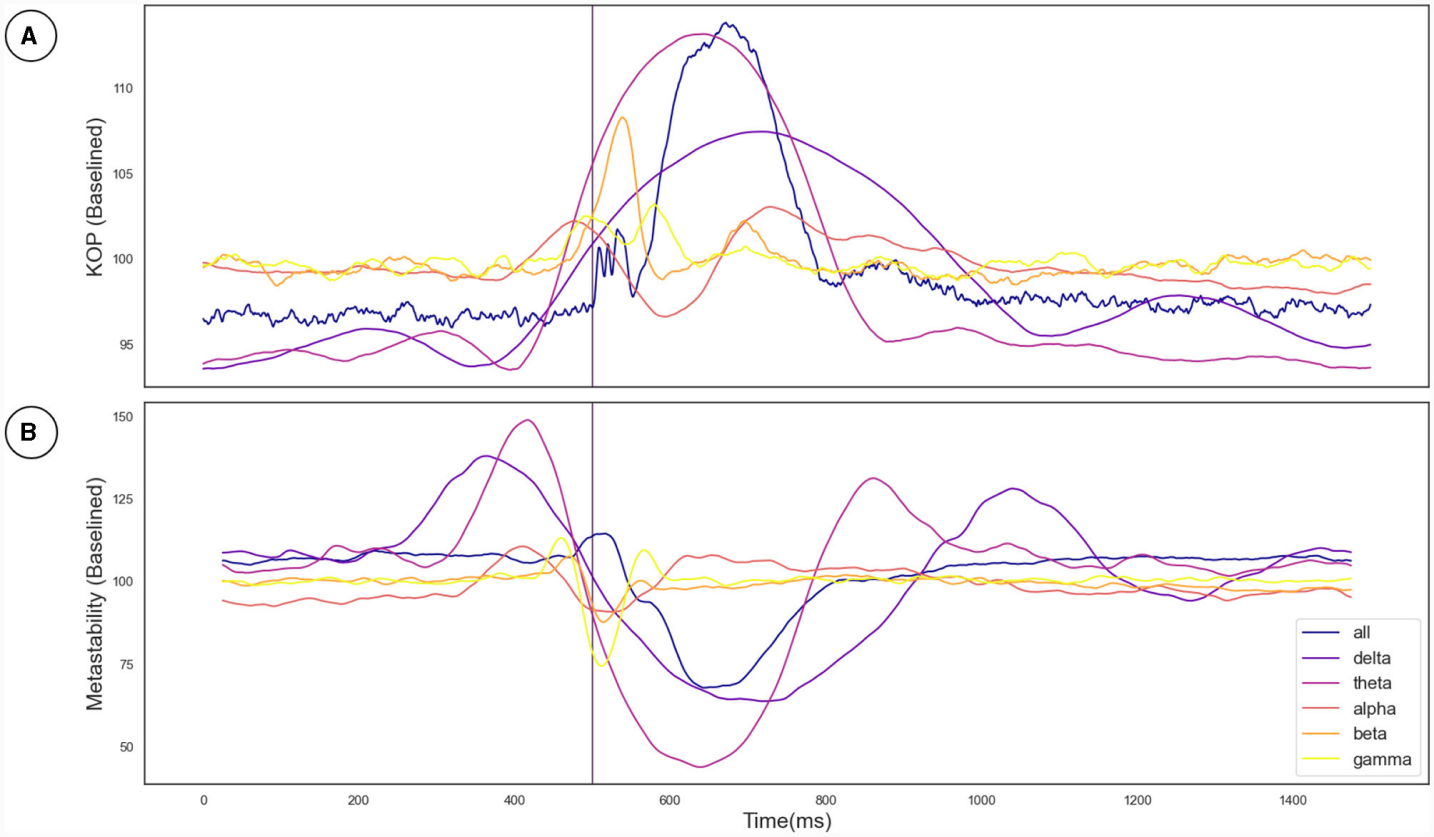
**(A)** The effect of the TMS pulse on the Kuramoto Order Parameter. **(B)** The effect of the TMS pulse on Metastability calculated in a sliding window. The TMS pulse is indicated by the vertical purple line at 500 ms. Both metastability and coherence are plotted as a percentage of a baseline value calculated as the mean between 525 and 1,525 ms. Results are plotted in unique colors for each frequency band as per the legend. These results pertain to the fronto-central electrode group and the 120% RMT stimulation condition as described in Section 3.2.

The Kuramoto Order Parameter was computed for the whole-brain scenario (all channels) and on the sub-groups of channels (fronto-central and occipito-temporal) to identify differences in the effect at the global and local level. Among all three groups, single pulse TMS causes a reduction of 10%–40% in the standard deviation of the KOP calculated in different frequency bands (see Section 2.3.1). Higher frequencies (alpha, beta and gamma) recover quickly (within 200 ms) while lower frequencies (delta and theta) recover slowly (within 400 ms). Interestingly, in all frequency bands, a 10% increase prior to the pulse is seen, while in the alpha and theta bands subsequent recovery 10%–15% past baseline levels was also observed. At a global level these changes were accompanied by a 10% decrease in KOP, however, for the subgroups, a sharp increase in KOP was observed concomitant with the same changes to metastability. These effects were replicated across all stimulation intensities, 120%, 110% and 120% of Resting Motor Threshold (RMT). The results for group 1 (fronto-central channels) and 120% RMT are plotted in Figure 3 and Table 1 details the significance of effects across different frequency bands. The same figures for the other channel groups and stimulation intensities can be found in the Supplementary material.

**Table 1:** Test statistics and *p*-values for one-tailed Wilcoxon's Signed Rank tests conducted on the Kuramoto Order Parameter measure (KOP).

	**Delta**	**Theta**	**Alpha**	**Beta**	**Gamma**	**All**
Decrease in KOP after pulse	200 (*p* < 0.001)	210 (*p* < 0.001)	88 (*p* = 0.73)	146 (*p* = 0.06)	202 (*p* < 0.001)	205 (*p* < 0.001)
Increase in KOP before pulse	6 (*p* < 0.001)	2 (*p* < 0.001)	21 (*p* < 0.001)	26 (*p* < 0.001)	13 (*p* < 0.001)	149 (*p* = 0.951)
Increase in KOP after pulse	162 (*p* = 0.985)	55 (*p* = 0.031)	17 (*p* < 0.001)	48 (*p* = 0.016)	82 (*p* = 0.204)	197 (*p* = 0.999)

**Figure 4:**
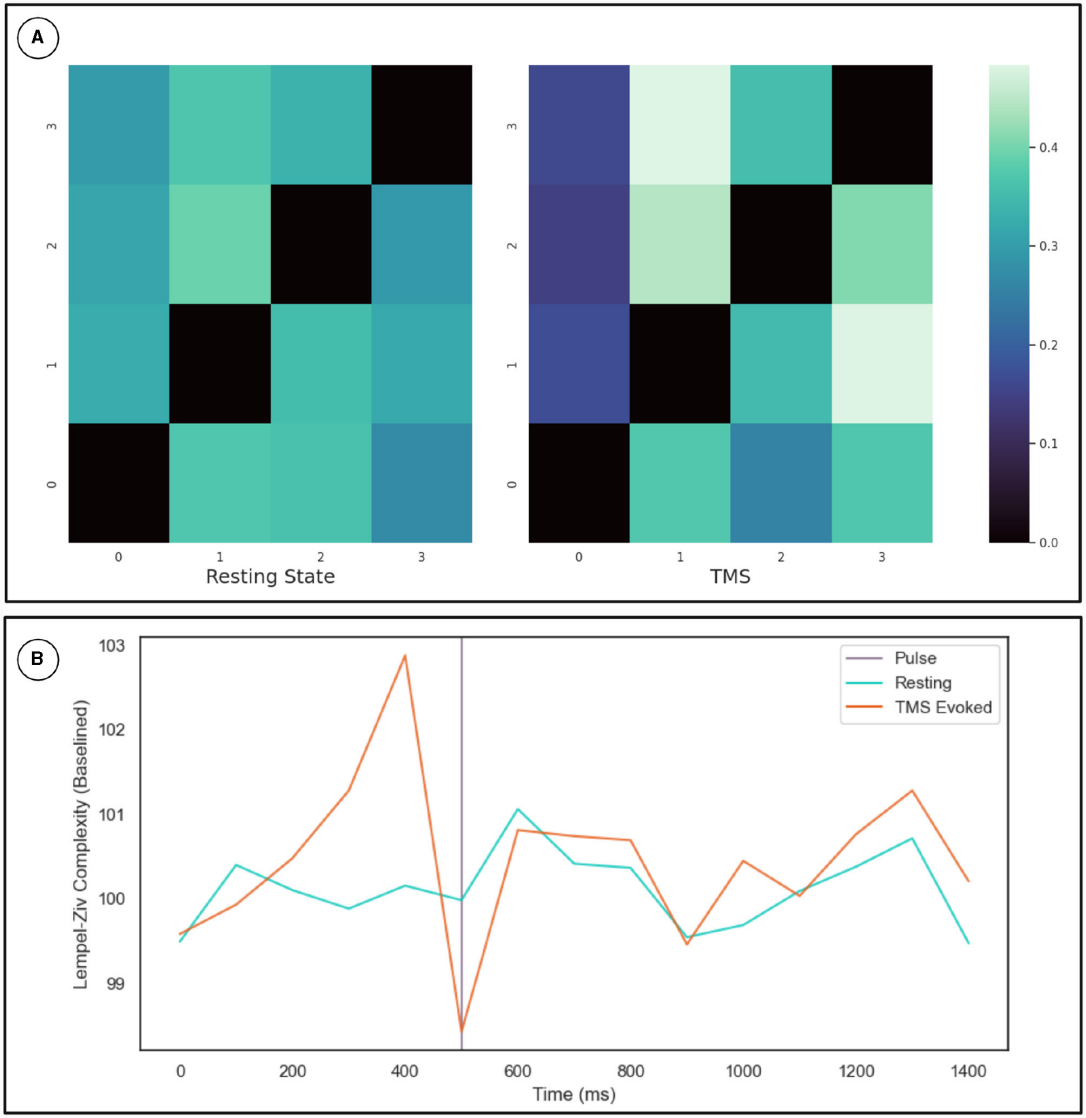
**(A)** Microstate transition probabilities. The numbers on the *x* and *y* axes indicate specific microstate. The probability of transitioning from the row microstate to the column microstate is given by the color of the cell as defined by the color map on the right. The left heat-map depicts transition probabilities for 1,000 ms of resting state data from a given participant. While the right heat-map depicts the same for an equivalent length of time following TMS stimulation. **(B)** Results for the Lempel-Ziv Complexity Measure. The TMS pulse is indicated by the purple line at 500 ms. The orange line is LZC in the TMS stimulated condition while the blue line is LZC in the resting state. Complexity was calculated in 100 ms bins and averaged across subjects and epochs. The resting state data was sub-sampled and averaged in the same way. Results are plotted as a percentage of the mean LZC across the first three bins.

To cross-validate the KOP measure of metastability, we calculated the Lempel-Ziv complexity of the sequence of microstates derived from resting state EEG data using a clustering algorithm (see Section 2.3.2). The optimal number of microstates varied across participants but was between 4 and 8 microstates in all cases. Microstate global explained variance was worse for the backfitted post-TMS EEG than for the resting state data but the performance was sufficient for continued analysis (0.65–0.60). Microstate analysis showed increased variation in microstate duration, and polarization of transition probabilities in the post-TMS data (Figure 4A). Note that the transition probabilities shown pertain to a single subject, since the subject specific microstate fitting made an averaging procedure unviable. However, the effect remains consistent across subjects. The LZC of the microstate sequence increases prior to the TMS pulse (test statistic = 22, *p* = 0.002), is reduced following TMS stimulation (test statistic = 138, *p*-value = 0.01) and recovers to baseline levels within 200 ms (Figure 4B).

The transition probabilities for the microstates were initially relatively consistent, with each microstate having a roughly even chance of transitioning to any other microstate. In the period after the TMS pulse for which metastability is reduced, transition probabilities increase along some columns and decrease along others. Since the columns indicate the probability of another microstate transitioning into a given microstate, this change reflects the repeated consolidation of dynamics to the same pattern(s) of activity.

### 3.3 Understanding post TMS modulation of metastability using a digital twin

**Figure 5:**
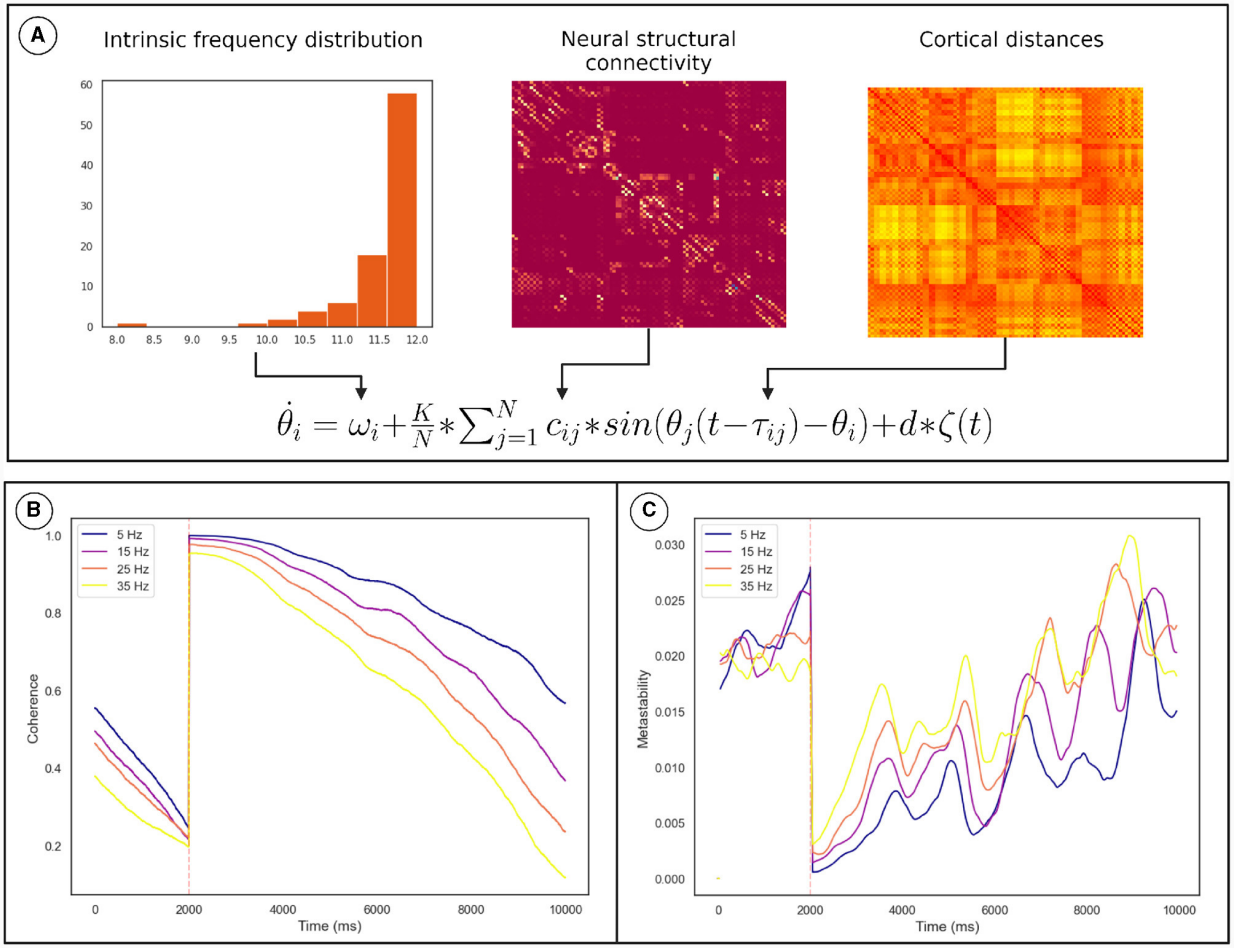
Overview of the computational model used to demonstrate the mechanism. **(A)** How the model parameters were derived from neural connectivity. **(B)** The effect of the phase reset on the The Kuramoto Order Parameter. **(C)** The effect of the phase reset on the metastability calculated in a sliding window. The orange line marks the time of stimulation. The results for each frequency distribution is plotted in a unique color whose highest frequency is described by the legend. Discontinuity at the pulse was removed for clarity. These results pertain to a subgroup of oscillators described in Section 2.4.

Numerical integration of the Kuramoto phase oscillators (Equation 2) connected via bio-physically realistic coupling parameters (*c*_*ij*_, τ_*ij*_; Figure 5A) was conducted using Euler integration in customized Python scripts. We identified the values of *K* and conduction velocity (that scales τ_*ij*_) for which the network is maximally metastable. The TMS pulse was simulated by resetting the phase of oscillators based on the conduction delay to the site of stimulation. The coherence and metastability was then computed from the simulated phase time series. The phase time series were simulated for multiple frequency distributions while keeping the other parameters constant (see Section 2.4).

Coherence increased following the TMS pulse followed by a reduction and recovery toward pre-TMS condition (Figure 5B). On the other hand, metastability decreases following TMS followed by a recovery toward baseline. Both of these findings were also observed in the empirical data. Furthermore, upon running the analysis for different maximum frequencies we observed that a quicker recovery of metastability occurs for higher frequency and slower recovery to baseline for lower frequency (Figure 5C).

## 4 Discussion

**Figure 6:**
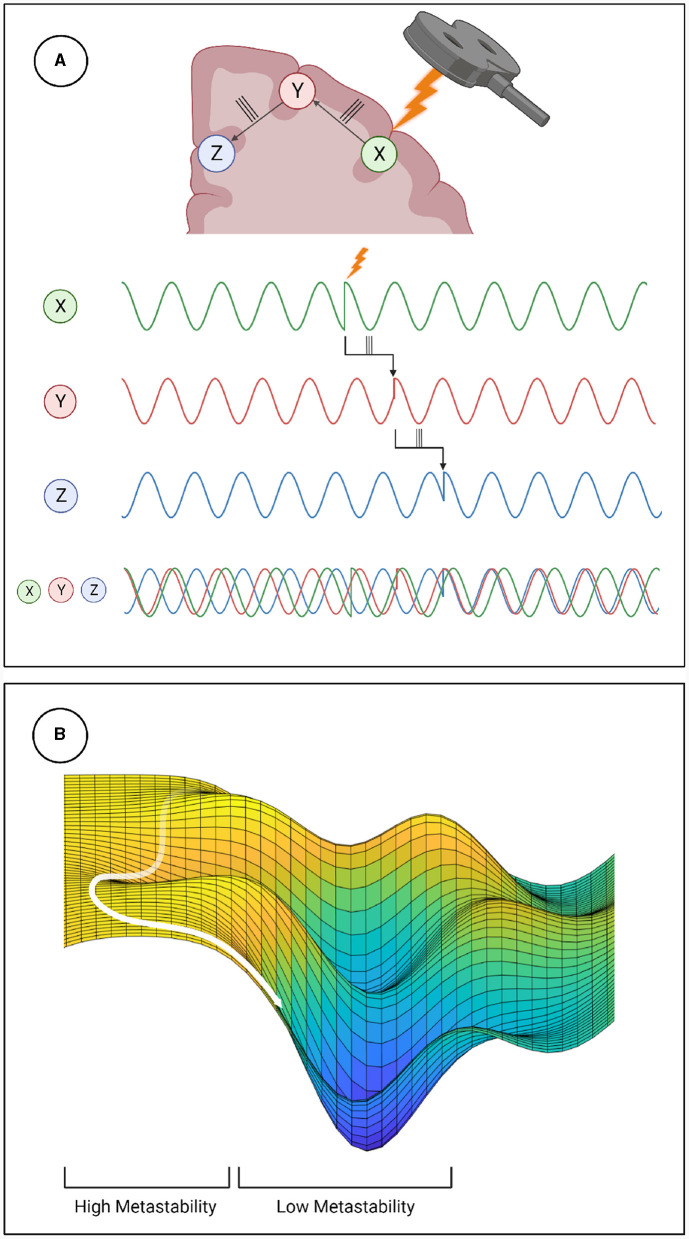
Mechanistic explanation of how TMS perturbs metastability. **(A)** The phase locking caused by TMS can propagate through the network, increasing coherence within clusters and decreasing the overall metastability. **(B)** Explanation of metastability in terms of an energy landscape. The system has high metastability when it moves in and out of points of stability (wells in the energy landscape) the period of depressed metastability is analogous to a valley, where movement is more constrained.

Metastability emerges when a delicate balance between integrative and segregative tendencies exists in a high dimensional large-scale network (Tognoli and Kelso, 2014). One can posit metastability as a winner-less competition between a set of coordination states (Deco et al., 2017). Emerging research have highlighted the usefulness of applying the measure of metastability for motor coordination (Tognoli and Kelso, 2014), lifespan aging (Naik et al., 2017), resting state brain dynamics (Deco et al., 2017) and mental health (Deco and Kringelbach, 2016). However, the changes in brain dynamics following brain stimulation techniques such as TMS, tDCS or tACS remains to explored in detail. Since the TMS pulse may lead to a forced synchronization certain groups of oscillators, it would follow that the natural balance between them would be disrupted and the dynamics would appear less metastable. Although this effect would be highly localized and brief due to the stimulation alone, the impulse delivered to the stimulated population could propagate through the network and reset their phase dynamics as it does so. This may effectively cluster neural populations based on their collective dynamics arising out of an interaction between synaptic weights and propagation delays. The relative simplicity and stability of the resulting coordination state would manifest in reduced metastability (see Figure 6). We proved this hypothesis in the present manuscript and validated our results using two independent measures of metastability, the standard deviation of Kuramoto Order Parameter (KOP) and Lempel-Ziv complexity (LZC; Figures 3, 4). We also observed a time-scale separation in recovery of metastability from higher frequency EEG components recovering faster than lower frequencies. Digging deeper to understand the mechanistic origins of this phenomenon we employed a coupled Kuramoto oscillator network using bio-physically realistic parameters. Both findings, metastability reduction and time scale separation of the recovery trajectory were replicated by the dynamical model giving us key insights to the network interactions that unfold during TMS.

Reduced Lempel-Ziv complexity following the TMS pulse corroborates the results of the KOP based analysis. Furthermore, following TMS, the microstate dynamics show increased transitions into certain microstates and reduced transitions into others. This polarization of the microstate transition probabilities suggests that the brain repeatedly consolidates to the same coupling state under reduced metastability. This aligns with how natural dynamics would periodically start to emerge but then be interrupted as the phase reset propagates through the network with finite time delays. The results of the modeling elucidates how reduced metastability post-TMS emerge from the dynamic interactions between structural parameters such as fiber width conduction speeds. Thus our approach demonstrates how a transient phase reset can produce a measurable reduction in metastability and the dissociation of its recovery in a tonotopic organization.

The validation of metastability as a measure of coordination dynamics in short timescales is the another contribution of this work. From a practical point of view this opens up opportunities for designing paradigms where an experimenter can choose to present task designs in a phase of reduced metastability if the effects of TMS has to be maximized. Alternatively, long term effects of TMS can be studied by presentation of stimulus at a temporal window where metastability has recovered. Furthermore, given how metastability is deranged in a wide range of neurological disorders (Hellyer et al., 2015; Córdova-Palomera et al., 2017; Cavanna et al., 2018; Lee et al., 2018), stimulation protocols aimed at treating them could show better effects if optimized based on their effect on metastability. It bears advantages over simpler measures such as coherence because of its relevance to pathology and its occurrence at multiple temporal and spatial scales. This property is exceptionally useful since it allows this measure to be applied to both EEG and MRI data, and capture changes occurring over disparate temporal and spatial scales.

An interesting and surprising result of this analysis was the increase in metastability observed prior to the pulse in the alpha and theta bands. Having thoroughly ruled out any artifactual sources of this effect, the most likely explanation was anticipation. Since hundreds of pulses were delivered during recording sessions, the distinctive “click” of the TMS coil was not masked and the inter-stimulus interval was consistent, it was possible that the subjects came to anticipate the pulse and the increase in metastability reflected that mental state. To address this question, the data were examined for the presence of a Stimulus Preceding Negativity (SPN). The SPN is an ERP associated with the anticipation of an affective or physiologically arousing stimulus such as opposite sex nudes or painful electric shocks (Luck and Kappenman, 2011). In one study, an electric shock was delivered 100–300 ms after an audio cue, and the SPN was observed in the time after the audio cue (Tanovic and Joormann, 2019). These conditions are highly reminiscent of the TMS coil click being followed by the scalp sensations of TMS. The presence of the SPN in the data (see Supplementary material) and the fact that TMS can produce sensations similar to electrical stimulation, supports the idea that participants were anticipating the stimulus in the same time window as the increase in metastability. The neurophysiological mechanism for this effect and whether the increase in metastability is facilitatory or collateral to anticipation is an area of future investigation. Paired and repetitive TMS are also known to produce long term effects on coherence and excitability which could alter metastability (Bharath et al., 2023).

Another intriguing implication of this work relates to how metastability changes during cognitive tasks. Metastability increases during cognitive tasks such as emotion perception, language processsing, and relational reasoning (Alderson et al., 2020). Additionally, decreases in metastability are associated with reduced cognitive flexibility (Hellyer et al., 2015). Thus a transient reduction in metastability could impair the performance of cognitive tasks. This could explain how TMS interferes with emotion perception (Pitcher et al., 2008), language processing (Devlin and Watkins, 2007) and relational reasoning (Ragni et al., 2016). Given that rTMS is known to have relatively long term effects, quantifying them using metastability and relating them to cognition is an interesting avenue for future research. Finally, the global desynchronization and local synchronization created by TMS presents a unique lens with which to analyse it's effects. Rather than seeing TMS as tool to excite one region, it might instead be seen as a way of linking a set of regions. Building a computational model of this phenomenon and using it to contextualize the effects of various TMS protocols is a direction of future study.

## Data availability statement

The original contributions presented in the study are included in the article/Supplementary material. The Python and MATLAB code used is available at the associated bitbucket repository: https://bitbucket.org/cbdl/metastabilitytms/src/master/.

## Ethics statement

Ethical approval was not required for this study as the data was acquired from publicly available repositories and the subjects were anonymized.

## Author contributions

RB: Data curation, Formal analysis, Investigation, Methodology, Software, Validation, Visualization, Writing – original draft, Conceptualization. AP: Methodology, Software, Writing – review & editing. AB: Conceptualization, Funding acquisition, Investigation, Methodology, Project administration, Resources, Supervision, Visualization, Writing – review & editing.
