# Supplementary material for: Metastability indexes global changes in the dynamic working point of the brain following brain stimulation
Source: Front Neurorobot. 2024 Feb 19;18:1336438. doi: 10.3389/fnbot.2024.1336438 (PMC10909933; doi:10.3389/fnbot.2024.1336438)
Supplement: Supplementary file 1 [file Data_Sheet_1.pdf]

Supplementary Material: Using metastability to identify the global changes in dynamic working point of the brain following brain stimulation

1 TMS ELECTRICAL FIELD SIMULATION

For the purpose of adding biophysical realism to the computational model, the region to be instantly phase reset by the simulated TMS was determined based on the electric field induced by the TMS pulse. SimNIBS (Saturnino et al., 2019) was used to simulate the electric field induced by a single pulse of TMS delivered to the rPMC using a MagStim D70 coil at 100% of RMT. RMT was determined based on the database created by Drakaki et al. (2022). The 95th percentile of field magnitudes induced, was considered the threshold for causing a phase reset. Another simulation was then done with the intensity at 120% of RMT. The regions where the electric field magnitude was greater than the aforementioned threshold were used for instant phase resetting.

2 EVENT RELATED POTENTIAL ANALYSIS

Event Related Potentials (ERPs) are electrical changes that are time locked to sensory, motor or cognitive events (Picton et al., 2000). They are obtained through averaging activity around an event across a large number of trials. To understand preliminary results showing changes in metastability prior to the TMS pulse, an analysis of ERPs was also performed. Specifically, slow negative waves, which are documented signs of anticipation were tested for using established procedures (Luck and Kappenman, 2011). This analysis consisted of low pass filtering the data at 2 Hz, epoching around the pulse and then averaging across epochs and participants. 200 milliseconds preceding the TMS pulse were then visually inspected for a slow negative wave.

The ERP analysis revealed a Stimulus Preceding Negativity (SPN) with a central distribution prior to the TMS pulse in all 3 stimulation intensities (see Figure S1). The small magnitude of this effect could be explained by signal attenuation due to the aggressive preprocessing pipeline.

3 SUPPLEMENTARY FIGURES

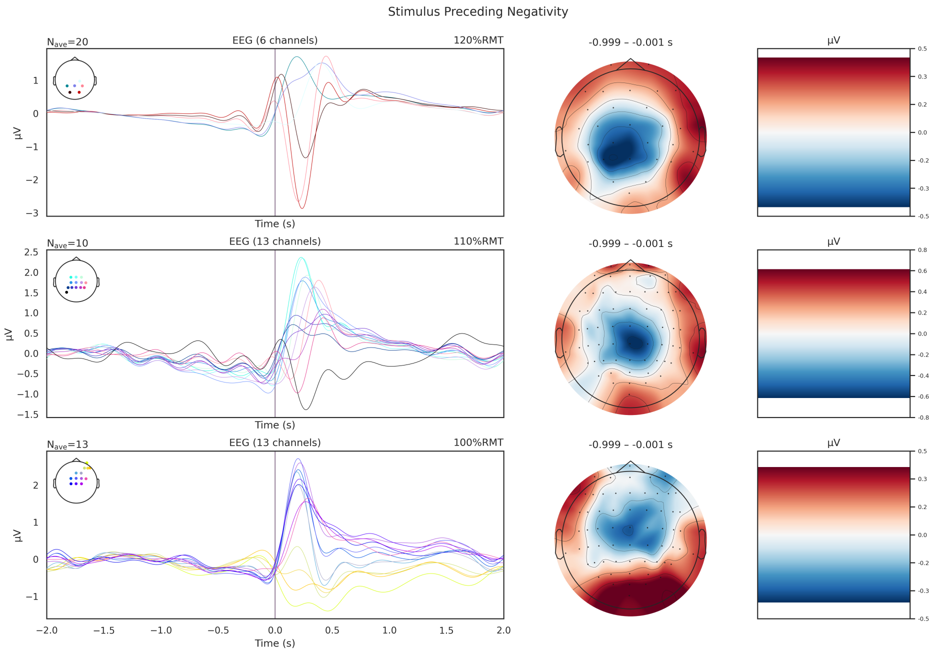

Figure S1. Stimulus Preceding Negativity (SPN) observed prior to the TMS pulse. Each row of plots pertains to a different stimulation intensity as indicated on the upper right of the ERP timeseries. The voltage distribution on the scalp is also shown indicating that the negativity is over the central electrodes.

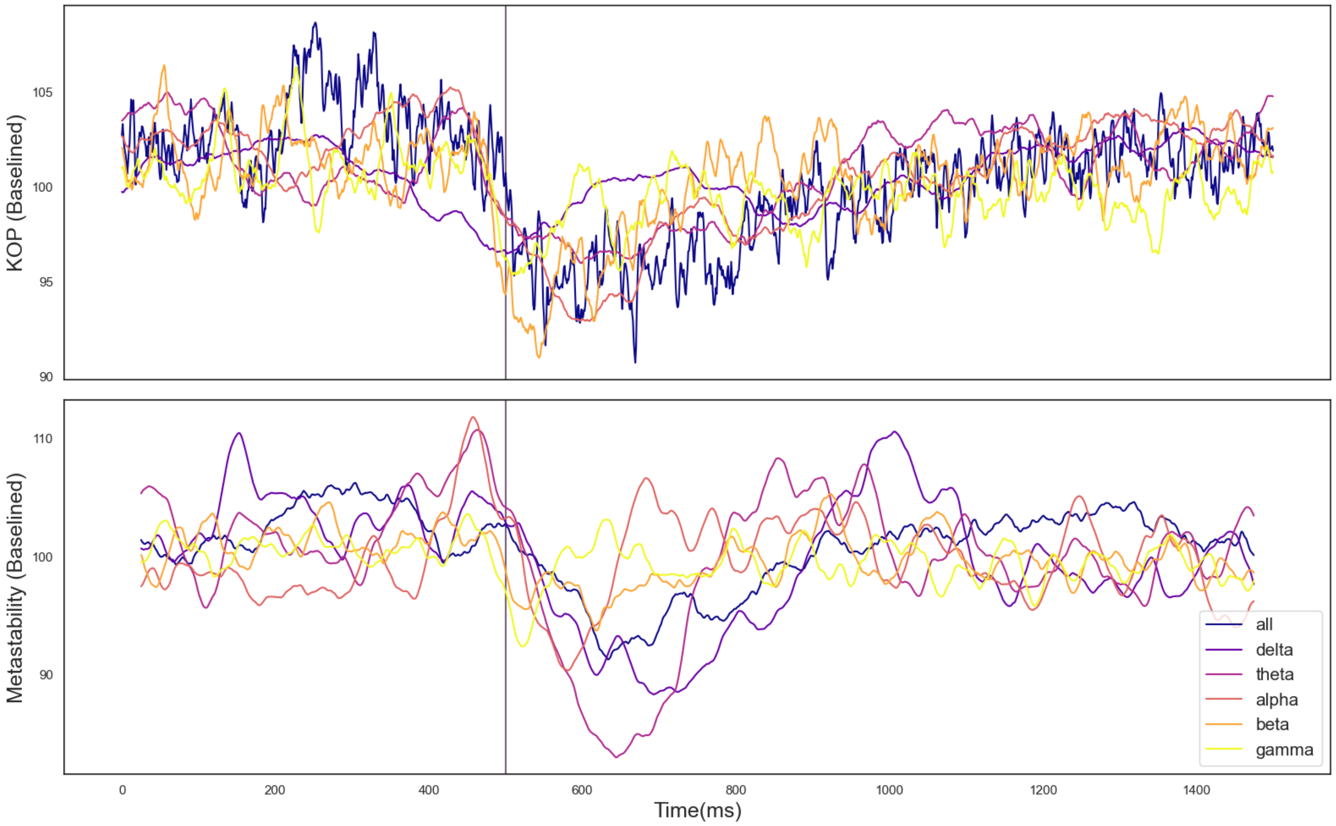

Figure S2. Results for all electrodes and the 100% RMT stimulation condition. On top is the effect of the TMS pulse on the Kuramoto Order Parameter. On the bottom is the effect of the TMS pulse on Metastability calculated in a sliding window. These results pertain to all electrodes and the 100% RMT stimulation condition. The TMS pulse is indicated by the vertical purple line. Both metastability and coherence are plotted as a percentage of a baseline value calculated as the mean between 525 and 1525 ms. Results are plotted in unique colors for each frequency band as per the legend.

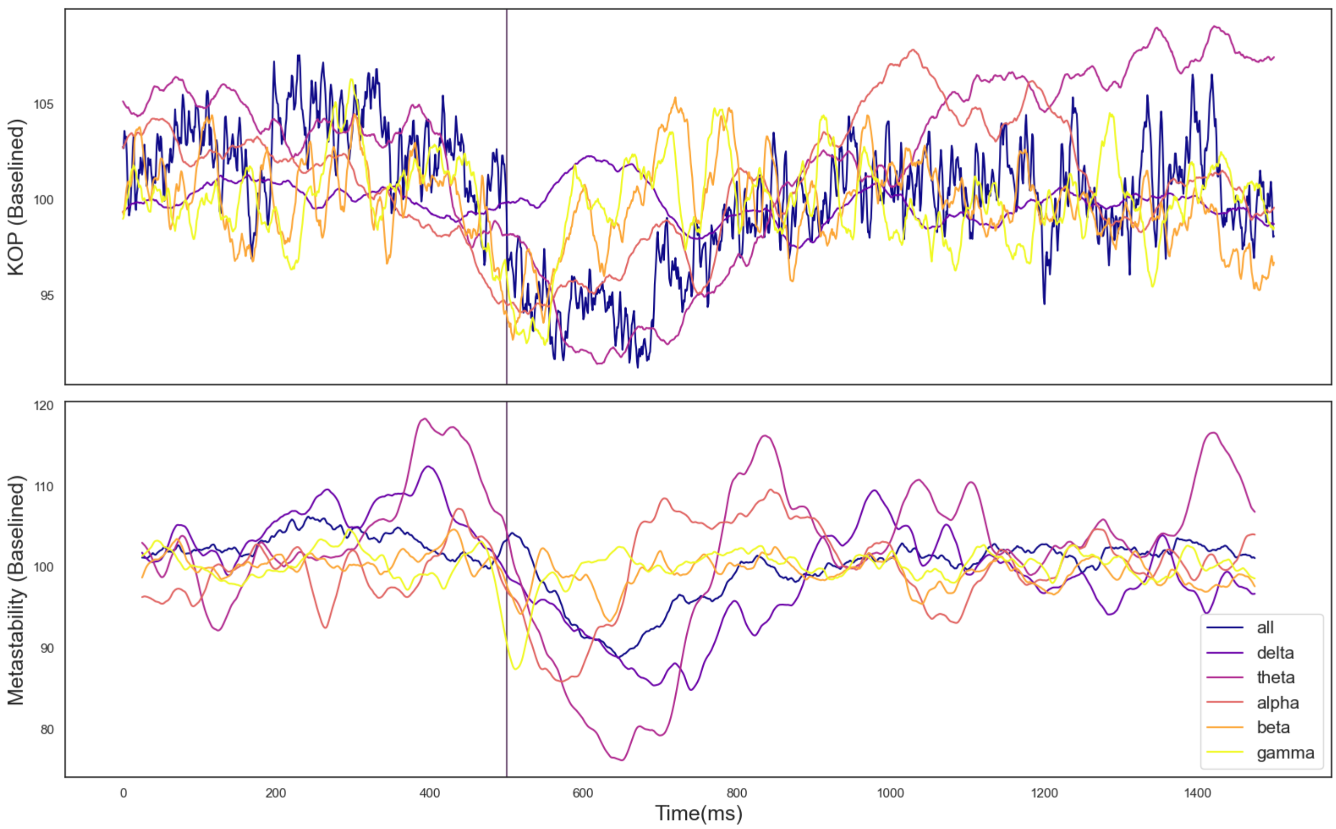

Figure S3. Results for all electrodes and the 110% RMT stimulation condition. On top is the effect of the TMS pulse on the Kuramoto Order Parameter. On the bottom is the effect of the TMS pulse on Metastability calculated in a sliding window. These results pertain to all electrodes and the 100% RMT stimulation condition. The TMS pulse is indicated by the vertical purple line. Both metastability and coherence are plotted as a percentage of a baseline value calculated as the mean between 525 and 1525 ms. Results are plotted in unique colors for each frequency band as per the legend.

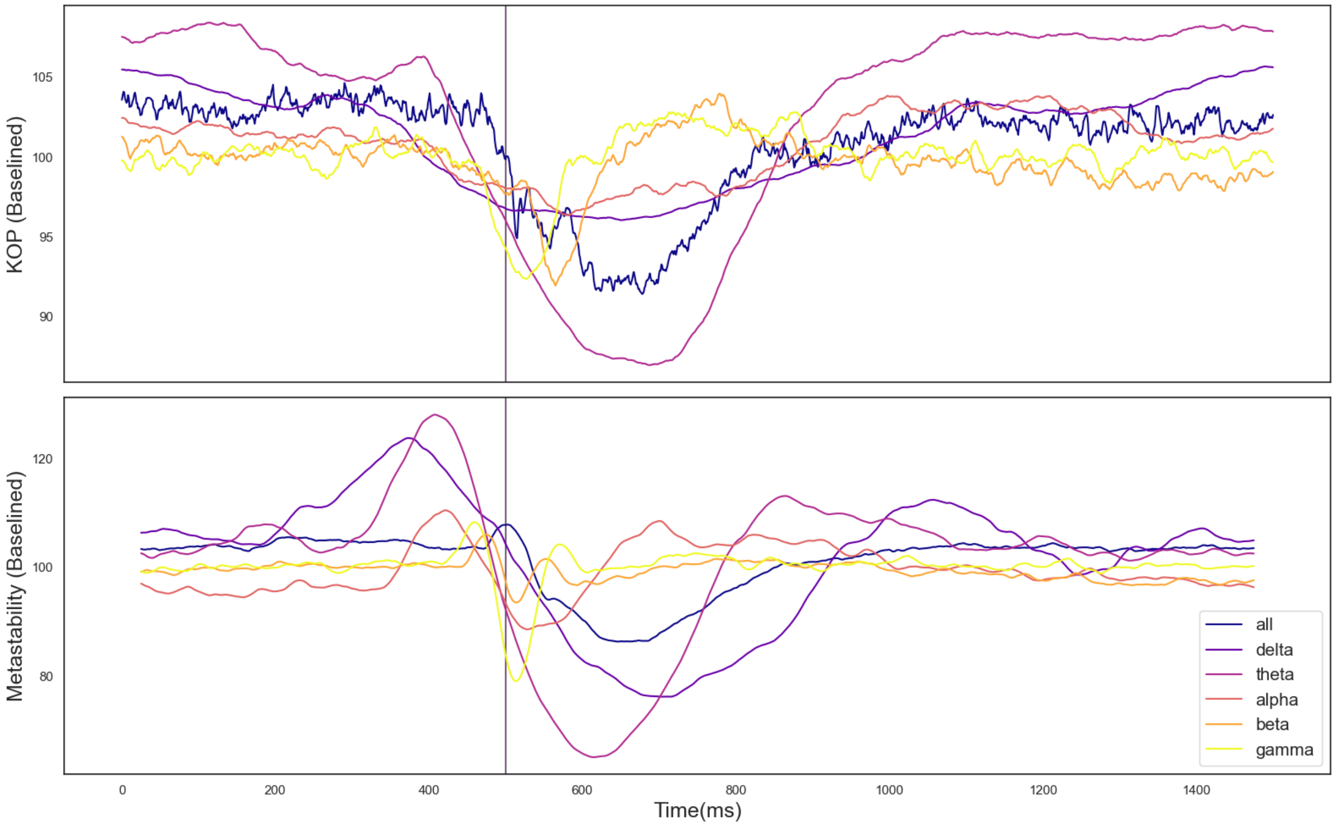

Figure S4. Results for all electrodes and the 120% RMT stimulation condition. On top is the effect of the TMS pulse on the Kuramoto Order Parameter. On the bottom is the effect of the TMS pulse on Metastability calculated in a sliding window. These results pertain to all electrodes and the 100% RMT stimulation condition. The TMS pulse is indicated by the vertical purple line. Both metastability and coherence are plotted as a percentage of a baseline value calculated as the mean between 525 and 1525 ms. Results are plotted in unique colors for each frequency band as per the legend.

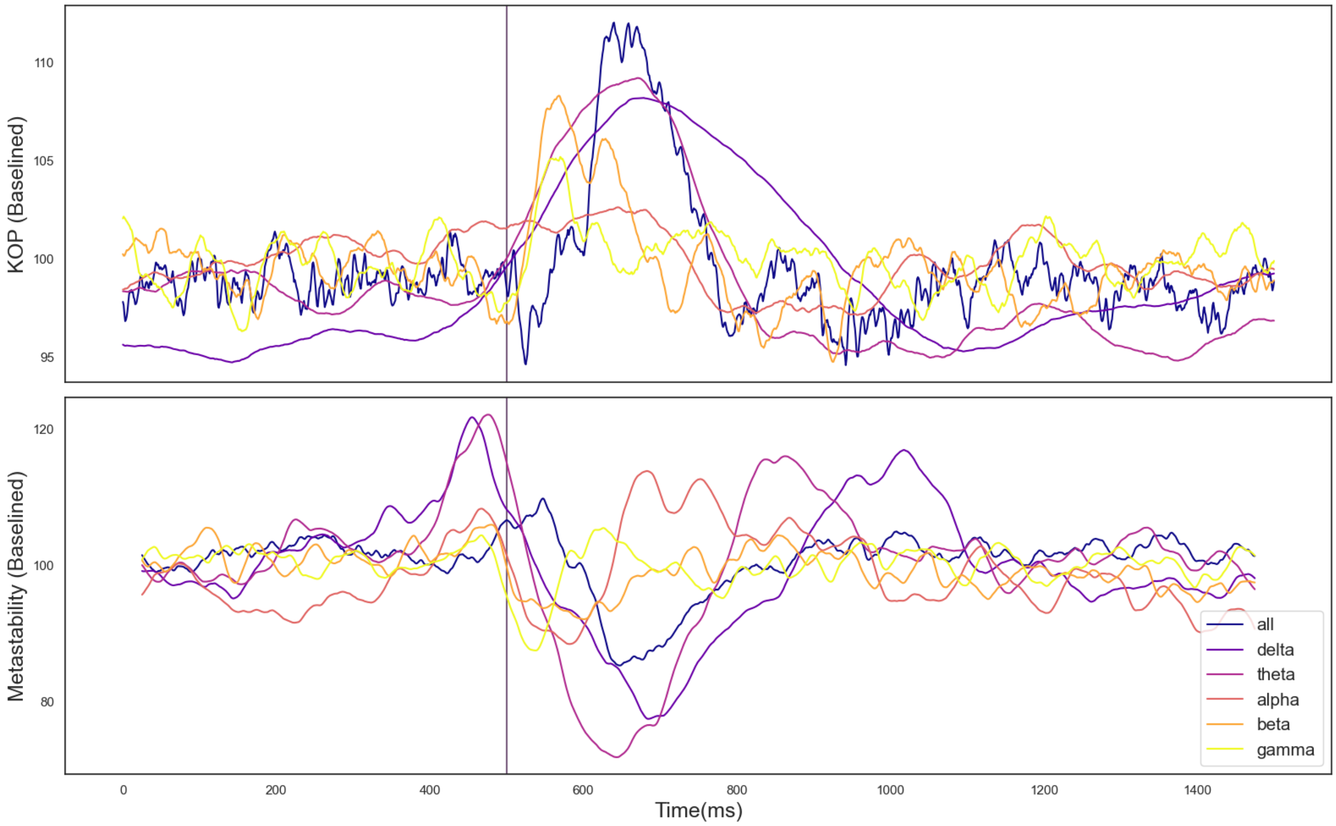

Figure S5. Results for the fronto-central electrode group and the 100% RMT stimulation condition. On top is the effect of the TMS pulse on the Kuramoto Order Parameter. On the bottom is the effect of the TMS pulse on Metastability calculated in a sliding window. These results pertain to all electrodes and the 100% RMT stimulation condition. The TMS pulse is indicated by the vertical purple line. Both metastability and coherence are plotted as a percentage of a baseline value calculated as the mean between 525 and 1525 ms. Results are plotted in unique colors for each frequency band as per the legend.

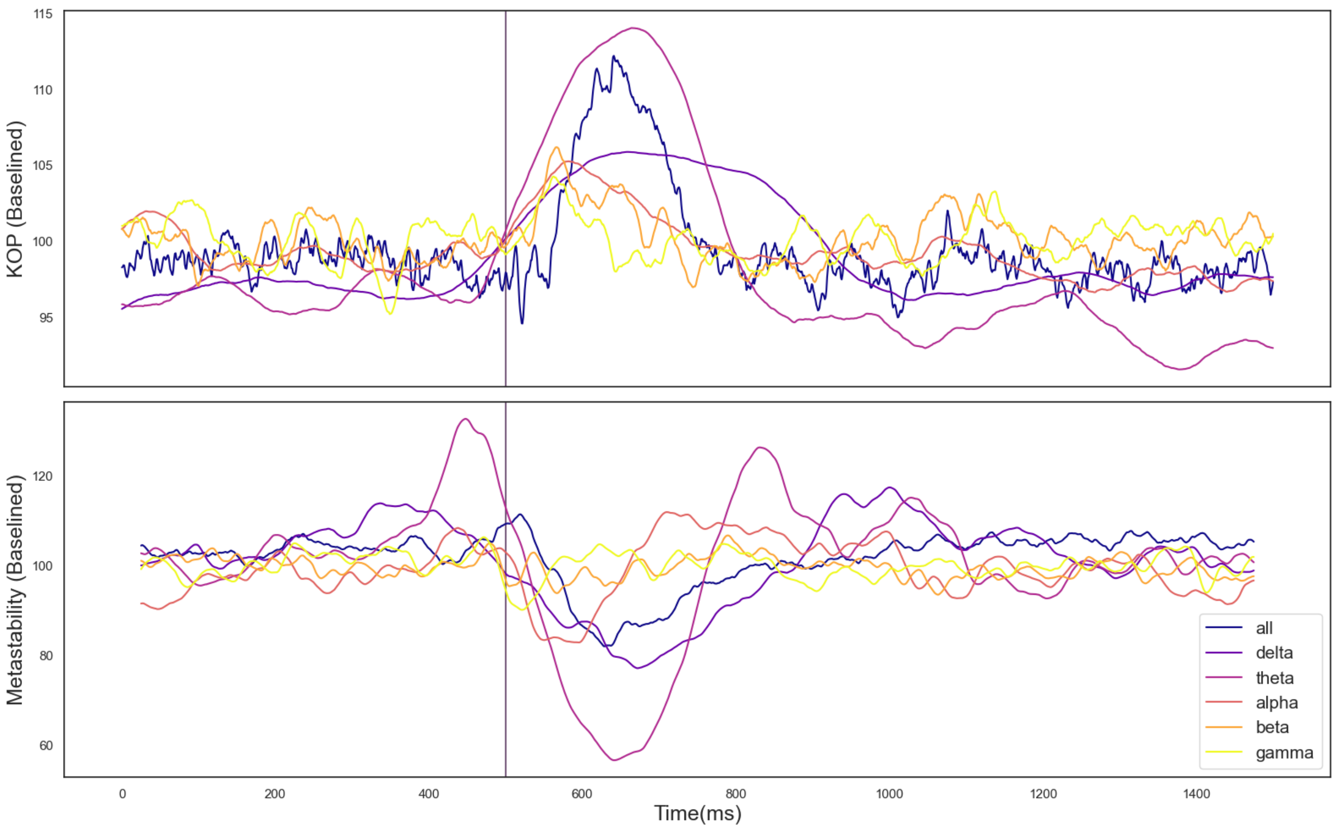

Figure S6. Results for the fronto-central electrode group and the 110% RMT stimulation condition. On top is the effect of the TMS pulse on the Kuramoto Order Parameter. On the bottom is the effect of the TMS pulse on Metastability calculated in a sliding window. These results pertain to all electrodes and the 100% RMT stimulation condition. The TMS pulse is indicated by the vertical purple line. Both metastability and coherence are plotted as a percentage of a baseline value calculated as the mean between 525 and 1525 ms. Results are plotted in unique colors for each frequency band as per the legend.

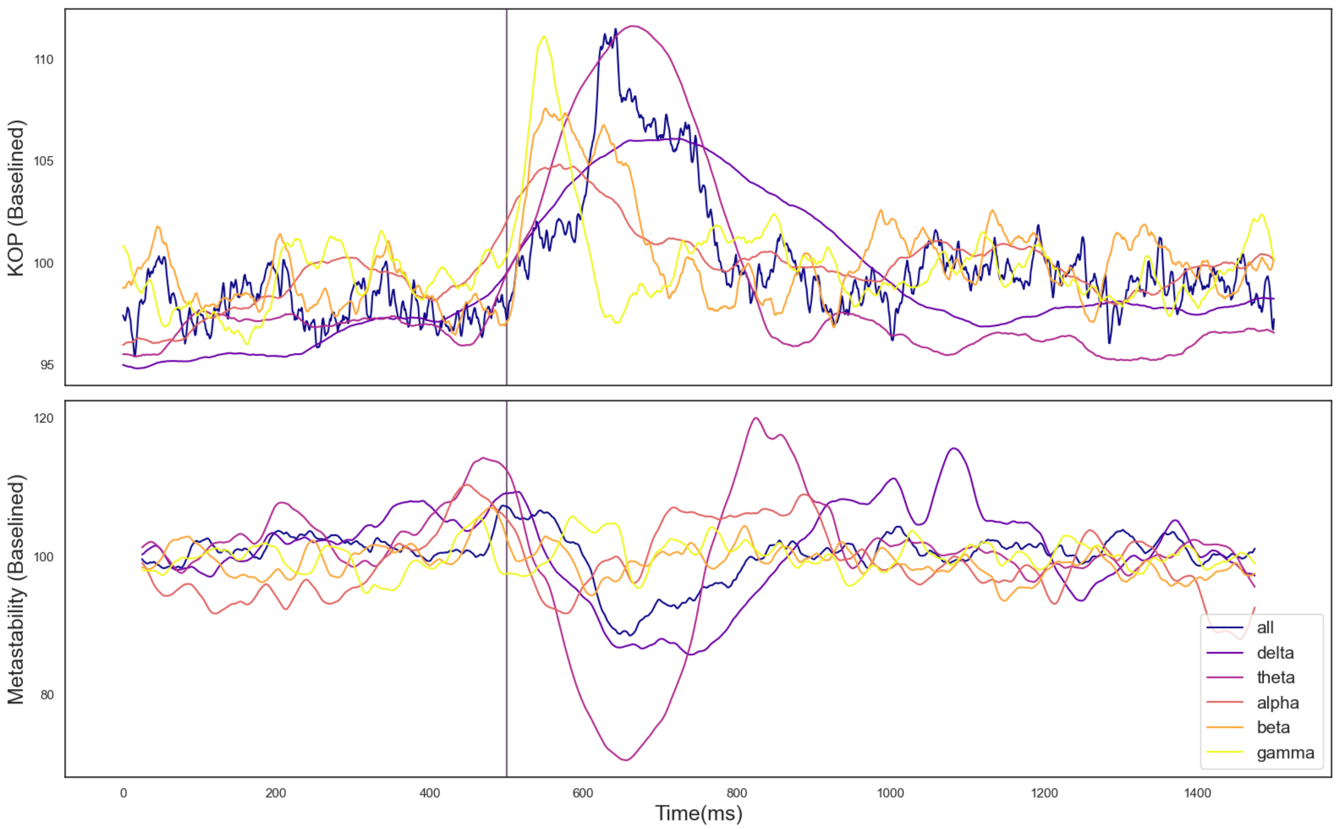

Figure S7. Results for the temporo-occipital electrode group and the 100% RMT stimulation condition. On top is the effect of the TMS pulse on the Kuramoto Order Parameter. On the bottom is the effect of the TMS pulse on Metastability calculated in a sliding window. These results pertain to all electrodes and the 100% RMT stimulation condition. The TMS pulse is indicated by the vertical purple line. Both metastability and coherence are plotted as a percentage of a baseline value calculated as the mean between 525 and 1525 ms. Results are plotted in unique colors for each frequency band as per the legend.

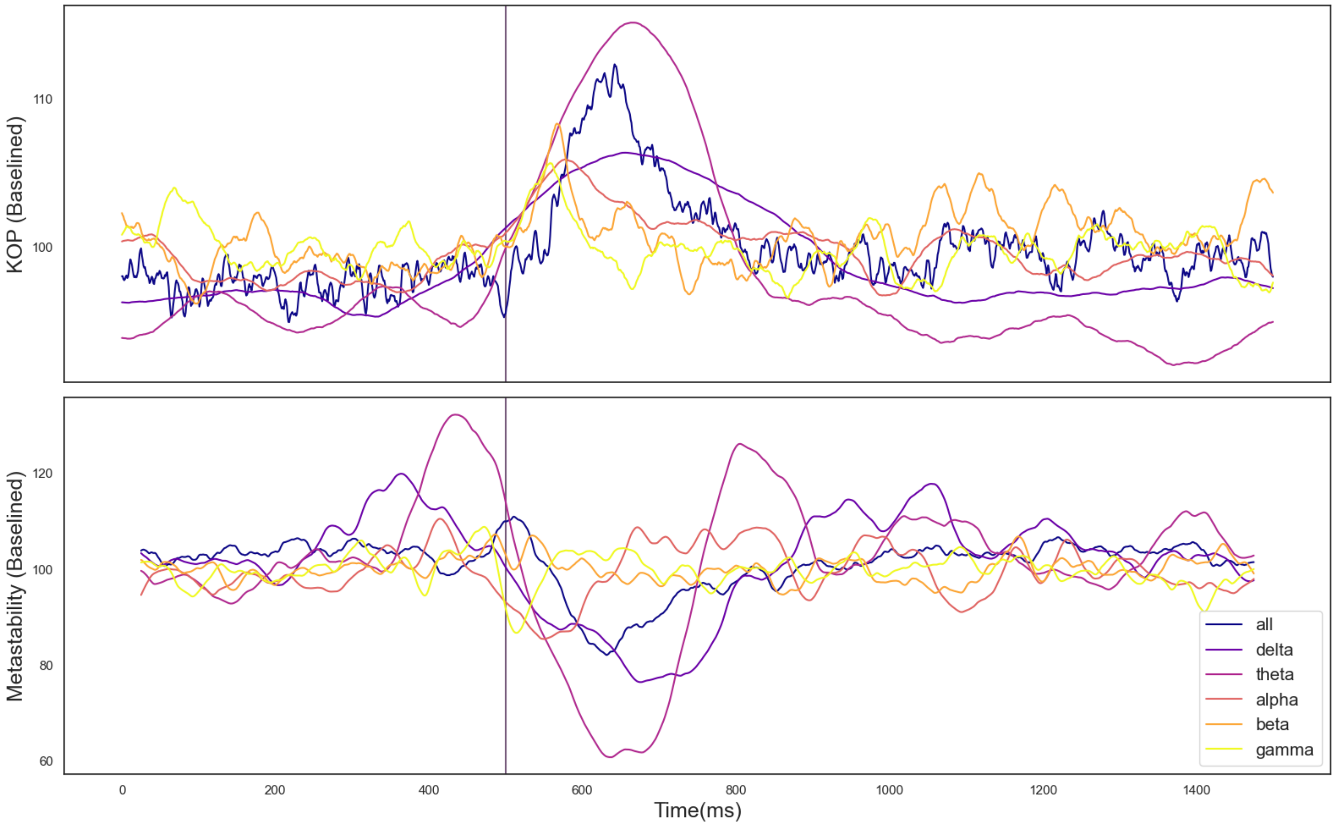

Figure S8. Results for the temporo-occipital electrode group and the 110% RMT stimulation condition. On top is the effect of the TMS pulse on the Kuramoto Order Parameter. On the bottom is the effect of the TMS pulse on Metastability calculated in a sliding window. These results pertain to all electrodes and the 100% RMT stimulation condition. The TMS pulse is indicated by the vertical purple line. Both metastability and coherence are plotted as a percentage of a baseline value calculated as the mean between 525 and 1525 ms. Results are plotted in unique colors for each frequency band as per the legend.

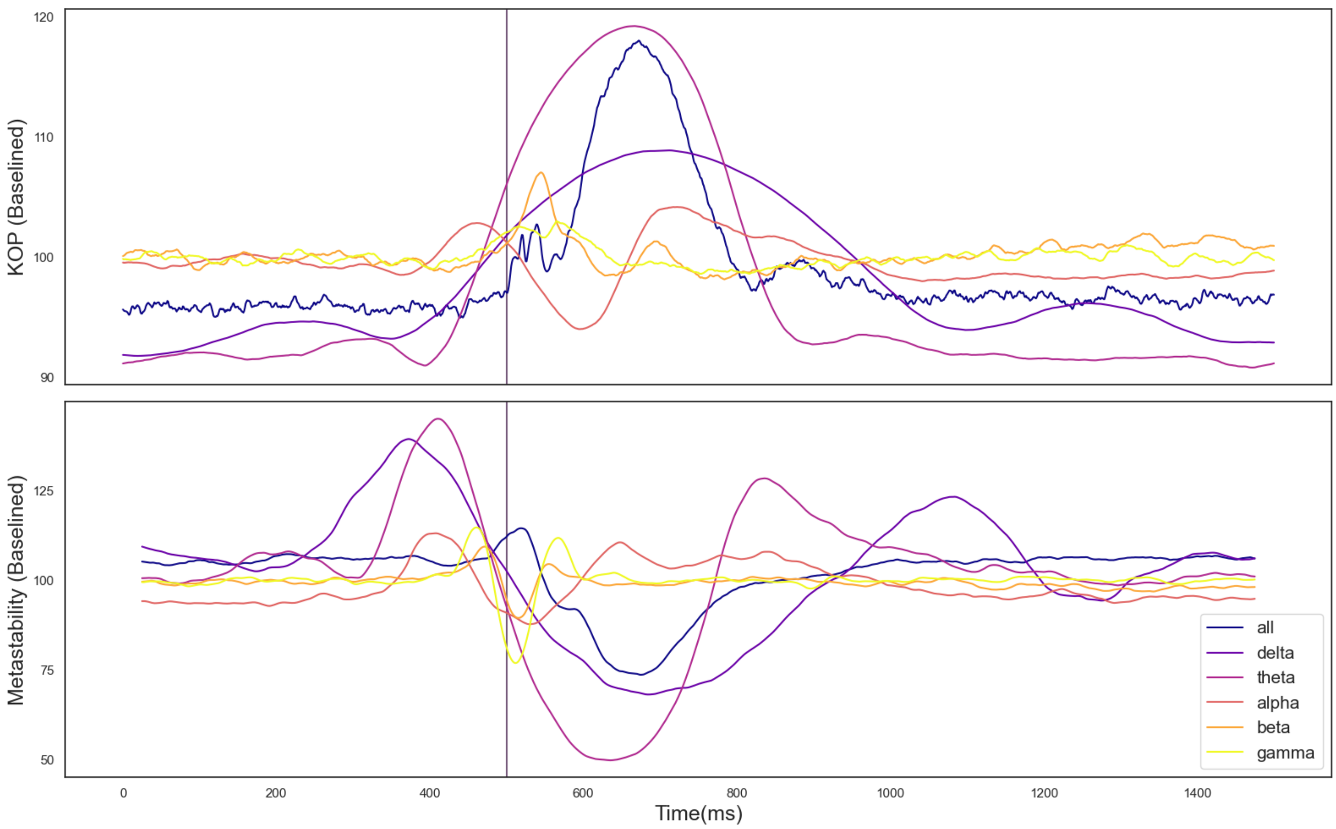

Figure S9. Results for the temporo-occipital electrode group and the 120% RMT stimulation condition. On top is the effect of the TMS pulse on the Kuramoto Order Parameter. On the bottom is the effect of the TMS pulse on Metastability calculated in a sliding window. These results pertain to all electrodes and the 100% RMT stimulation condition. The TMS pulse is indicated by the vertical purple line. Both metastability and coherence are plotted as a percentage of a baseline value calculated as the mean between 525 and 1525 ms. Results are plotted in unique colors for each frequency band as per the legend.

REFERENCES

Maria Drakaki, Claus Mathiesen, Hartwig R. Siebner, Kristoffer Madsen, and Axel Thielscher. Database of 25 validated coil models for electric field simulations for tms. *Brain Stimulation*, 15(3):697–706, 2022. ISSN 1935-861X. doi: 10.1016/j.brs.2022.04.017.

Steven J. Luck and Emily S. Kappenman. *The Oxford Handbook of Event-Related Potential Components*. Oxford University Press, December 2011. ISBN 9780199705870. Google-Books-ID: gItoAgAAQBAJ.

T. Picton, S. Bentin, P. Berg, E. Donchin, S. Hillyard, Ray Johnson, Gregory A. Miller, W. Ritter, D. Ruchkin, M. Rugg, and Margot J. Taylor. Guidelines for using human event-related potentials to study cognition: recording standards and publication criteria. 2000. doi: 10.1111/1469-8986.3720127. URL `https://www.semanticscholar.org/paper/4297e34b99c7a65917a139bec56e6325d6e3585d`.

Guilherme B. Saturnino, Oula Puonti, Jesper D. Nielsen, Daria Antonenko, Kristoffer H. Madsen, and Axel Thielscher. Simnibs 2.1: A comprehensive pipeline for individualized electric field modelling for transcranial brain stimulation, 2019.
